# Further analysis of the lens of ephrin-A5^−/−^ mice: development of postnatal defects

**Published:** 2013-02-03

**Authors:** Alexander I. Son, Margaret A. Cooper, Michal Sheleg, Yuhai Sun, Norman J. Kleiman, Renping Zhou

**Affiliations:** 1 Department of Chemical Biology, Susan Lehman-Cullman Laboratory for Cancer Research, Ernest Mario School of Pharmacy, Rutgers University, Piscataway, NJ; 2 Department of Molecular Biophysics and Biochemistry, Yale University, New Haven, CT; 3 Department of Environmental Health Sciences, Mailman School of Public Health, Columbia University, New York, NY

## Abstract

**Purpose:**

The cells of the mammalian lens must be carefully organized and regulated to maintain clarity. Recent studies have identified the Eph receptor ligand ephrin-A5 as a major contributor to lens development, as mice lacking ephrin-A5 develop abnormal lenses, resulting in cataracts. As a follow-up to our previous study on the cataracts observed in ephrin-A5^−/−^ animals, we have further examined the morphological and molecular changes in the ephrin-A5^−/−^ lens.

**Methods:**

Wild-type and ephrin-A5^−/−^ eyes at various ages were fixed, sectioned, and examined using histological techniques. Protein expression and localization were determined using immunohistochemistry and western blot analysis.

**Results:**

Lens abnormalities in the ephrin-A5^−/−^ animals are observed at postnatal stages, with lens opacity occurring by postnatal day 21. Structural defects in the lens are first observed in the outer lens fiber cell region where cells in the ephrin-A5^−/−^ lens are severely disorganized. Ephrin-A5 and the Eph receptor EphA2 are expressed during early ocular development and continue to be expressed into postnatal stages. The cataracts in the ephrin-A5^−/−^ mutants occur regardless of the presence of the CP49 mutation.

**Conclusions:**

In this follow-up study, we have uncovered additional details explicating the mechanisms underlying ephrin-A5 function in the lens. Furthermore, elucidation of the expression of ephrin-A5 and the Eph receptor EphA2 in the lens supports a fundamental role for this receptor-ligand complex in lens development. These observations, in concert with our previous study, strongly suggest that ephrin-A5 has a critical role in postnatal lens fiber organization to maintain lens transparency.

## Introduction

The mammalian lens requires precise cellular organization to maintain clarity throughout the lifetime of the animal. The lens is an asymmetric spheroid with unique anatomy containing a single epithelial layer on its anterior surface and underlying rows of fiber cells arranged in precisely arranged layers. The fiber cells consist of two distinct populations: embryonic (primary) fiber cells forming the nuclear core of the lens, and the cortical (secondary) fiber cell layers overlying the primary cell layer [1,2]. The embryonic fiber cell layer is formed during the earliest stages of lens development, while the cortical fiber layers surrounding this core are derived from dividing and differentiating lens epithelial cells located near the lens equator. Throughout life, new lens fiber cells are added onto the lens [3]. This continual process of differentiation by the lens epithelium into fiber cells is marked by unique alterations in cell morphology, as cells become elongated along the anterior-posterior axis. In addition, lens fiber cells undergo distinct changes in cytoplasmic profile, including organelle degradation and the expression of crystallin proteins, to maintain structure and stability [4-9].

The postnatal lens is an avascular tissue that receives nourishment from its surrounding aqueous and vitreous fluids [10,11]. Because of this lack of vasculature, it is believed that intercellular interactions and communication between the fiber cells is integral to lens function and is under tight regulatory control [10,11]. In cross-section, elongated fiber cells are packed into flattened hexagons of uniform size along meridional rows [12]. This architecture is maintained by several known cell adhesion mechanisms. One major family of molecules regulating cell-cell adhesion is the adherens junction complex, which contains the calcium-dependent homophilic-binding molecules termed cadherins [13]. The lens contains two major populations of cadherins: E-cadherin expressed exclusively in the lens epithelium and N-cadherin in the lens fiber cells [14-17]. Also critical are the functions of gap junctions, channel structures composed of connexin proteins that allow the passage of small molecules between adjacent cells [18-20]. The lens contains three major connexin molecules: connexin 43 (Cx-43) expressed in the lens epithelium, connexin 46 (Cx-46) located exclusively in the differentiated fiber cells, and connexin 50 (Cx-50) in both the epithelium and fiber cell populations [20]. Aquaporin 0 (AQP0), the most abundant membrane protein in lens fiber cells responsible for passive water diffusion [21,22], has also recently been shown to play important roles in fiber cell adhesion [23,24]. However, while the factors responsible for intercellular interactions have been well documented, the mechanisms regulating these interactions remain unclear.

Recently, the Eph family of receptor tyrosine kinases has been found to play a critical role in lens development and maturation (as reviewed in [25]). This group of molecules consist of the Eph receptors, with 16 members grouped into the EphA and EphB subclasses, and the ephrin ligands, which contain eight molecules divided into the glycosylphosphatidylinositol-anchored ephrin-A and the transmembrane-spanning ephrin-B subclasses [26,27]. Eph receptors of one subclass interact with most if not all ephrin ligands of the same subclass, although interactions between differing receptor and ligand subclasses also occur [28-30]. The signaling events regulated by the ephrins have been implicated in many biological roles ranging from nervous system development to vascular development [27].

We and others have shown previously that the ligand ephrin-A5 and receptor EphA2 both play critical roles in normal lens function, as both ephrin-A5^−/−^ and EphA2^−/−^ mice develop cataracts [31-34]. In addition, the human homolog EPHA2 has been linked with congenital cataracts in patients [33,35-38]. However, while the importance of this family of molecules in lens function has been firmly established, the specific roles these molecules play in lens development remain unclear. We have previously reported major deformities in the ephrin-A5^−/−^ mouse lens fiber cell layers leading to cataract formation [32]. In contrast, another study identified the ephrin-A5^−/−^ lens abnormalities to be restricted to only the lens epithelium with minimal changes in the lens fiber cell layer [31]. To follow up our former study, we have further investigated the function of ephrin-A5 in the lens by identifying its expression during development and determining the spatial and temporal changes that lead to cataracts in ephrin-A5^−/−^ mice. In addition, we have examined potential interactions with the CP49 mutation and found that ephrin-A5 functions independently of CP49.

## Methods

### Animals

Mice were bred and cared for in accordance with the Guidelines for the Care and Use of Laboratory Animals of Rutgers University and the Association for Research in Vision and Ophthalmology Statement for the Use of Animals in Ophthalmic and Vision Research. Ephrin-A5^−/−^ mice [39] and EphA2^LacZ/LacZ^ mice [33] have been previously described. CP49 status was determined using previously established methods [40]. Briefly, isolated genomic DNA from tail tissue was prepared for PCR at a volume of 50 μl under the following conditions: 1x PCR buffer, 25 mM MgCl_2_, 0.1 mM deoxyribonucleotide triphosphate (dNTP) mix, 0.5 μM of each primer, 0.625 U Taq DNA polymerase (New England Biolabs, Ipswich, MA), and 2 μl (100 to 200 ng) of total DNA. Wild-type CP49 was determined using primers e (5′-TTG GAA ACA ACC TCC AGA CCA GAG-3′) and c’ (5′-ACA TTC TAT TTC GAG GCA GGG TCC-3′), producing a 403 bp product, while mutant CP49 with the 6 kb deletion was determined using primers c (5′-TGG GGT TGG GCT AGA AAT CTC AGA-3′) and e’ (5′-AGC CCC TAC GAC CTG ATT TTT GAG-3′), producing a 386 bp product [40].

### Mouse lens imaging

For imaging of whole mount lenses, mouse eyes were enucleated and lenses dissected in prewarmed Dulbecco’s Modified Eagle Medium (DMEM; Sigma-Aldrich, St. Louis, MO) at 37 °C over a mesh grid. Imaging was performed using a Nikon SMZ 1500 microscope.

### Hematoxylin and eosin staining

Embryos and postnatal eyes were prepared using lens fixation buffer (65% ethanol, 4% formaldehyde, 5% acetic acid, 3% sucrose) at 4 °C, dehydrated, and embedded in Paraplast (McCormick Scientific). Longitudinal sections were prepared at 5 μm and stained with hematoxylin and eosin (Sigma-Aldrich).

### Immunohistochemistry

Postnatal eyes were enucleated and fixed in 4% formaldehyde for 10 min at room temperature, followed by a rinse in 0.01 M phosphate buffered saline (PBS; 0.138 M NaCl, 0.0027 M KCl) for 5 min, and stored in 10% sucrose overnight at 4 °C. Tissues were subsequently frozen and cryosectioned at 10 μm.

For lens epithelial whole mounts, postnatal eyes were enucleated and lenses carefully dissected out. The lens capsule was then carefully removed with its anterior epithelial layer attached. Epithelial whole mounts were fixed in 4% formaldehyde for 10 min at room temperature, followed by a rinse in PBS for 5 min before immunostaining.

Lens tissue was stained using antibodies against β-catenin (1:3,000, BD Biosciences; San Jose, CA), E-cadherin (1:1,000, BD Biosciences), and zona occludens 1 (ZO-1; 1:200, Invitrogen, Grand Island, NY). The antibody against AQP0 (1:1,000) was generously provided by Dr. J. Samuel Zigler. Secondary antibodies used include Alexa Fluor 488 conjugated antimouse immunoglobulin G (IgG; 1:200, Invitrogen), Alexa Fluor 488-conjugated antirabbit IgG (1:200, Invitrogen), and CY3-conjugated antimouse IgG (1:200, Jackson ImmunoResearch Laboratories, Inc., West Grove, PA).

### Lens suture analysis

Analysis of the posterior suture was done using a modified protocol as reported by Shi et al. [41]. Enucleated eyes were dissected and lenses were removed in prewarmed DMEM at 37 °C. The lens capsule was then carefully removed, and the tissue was incubated in 1 μM FM4–64 styryl dye (Invitrogen, F34653) in prewarmed DMEM. Decapsulated lenses were incubated in the dye for 15 min before imaging. Subsequent confocal images were taken in the presence of the FM4–64 dye.

### Detection of Ephrin-A protein expression using EphA5–alkaline phosphatase

The EphA5–alkaline phosphatase (AP) affinity probe for ephrin ligand detection has been described previously [42-44]. To assess ephrin ligand and Eph receptor binding, frozen tissue sectioned at 14 μm was mounted and quickly dried with a blow dryer. Sections were fixed in 4% paraformaldehyde in PBS for 8 min at room temperature, followed by two washes in PBS for 5 min each. Concentrated medium containing EphA5-AP was then applied to the sections for 2 h followed by washes in Hanks’ balanced salt solution with 0.5 mg/ml bovine serum albumin and 20 mM HEPES (pH 7.0). Sections were fixed again in 60% acetone, 3% formaldehyde, and 20 mM HEPES (pH 7.5) for 30 s, followed by two washes in wash buffer (150 mM NaCl and 20 mM HEPES [pH 7.5]) for 5 min each. Sections were then heated to 65 °C for 15 min and washed again in wash buffer, followed by a rinse in AP Color Development Buffer (100 mM Tris-HCl [pH. 9.5], 100 mM NaCl, 5 mM MgCl_2_). Color development was achieved by incubating sections in AP Color Development Buffer with nitro blue tetrazolium/5-bromo-4-chloro-3-indolyl-phosphate (NBT/BCIP) solution (1:50 [0.17 mg/ml BCIP, 0.33 mg/ml NBT] Roche Indianapolis, IN, 11,681,451,001) at room temperature until sections were sufficiently stained.

### β-Galactosidase staining

Embryos and eyes were fresh frozen and cryosectioned at 12 μm. Sections were post-fixed in a 2% paraformaldehyde/0.5% glutaraldehyde solution in 1x PBS for 1 min, followed by a rinse in 1x PBS for 5 min. Samples were then incubated in reaction buffer (1 mg/ml 5-bromo-4-chloro-3-indolyl-β-galactopyranoside [X-Gal], 5 mM potassium ferricyanide, 5 mM potassium ferrocyanide, 2 mM magnesium chloride, 0.01% sodium deoxycholate, and 0.02% Nonidet P-40 [NP-40]) for 18 h at 37 ºC. Detailed methods for β-galactosidase staining has been described previously [45].

## Results

### Alterations in gross morphology of the postnatal ephrin-A5^−/−^ lens

**Figure 1:**
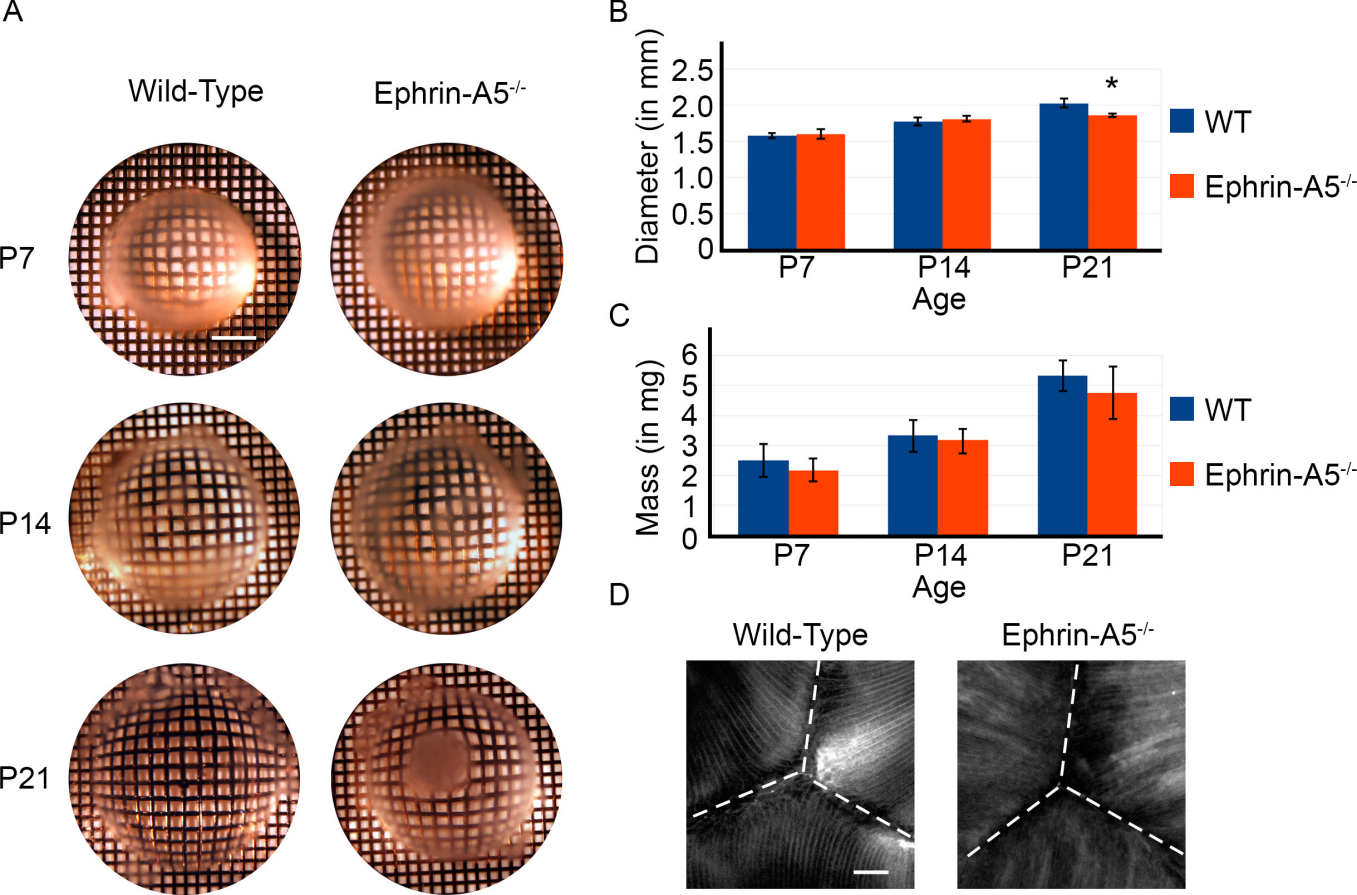
Disruptions in the gross morphology of the ephrin-A5^-/-^ lens appear in postnatal stages. **A**: Postnatal day 7 (P7) and P14 ephrin-A5 mutant lenses appear grossly normal. However, by P21 opacity becomes quite prominent in the ephrin-A5^-/-^ lens while the wild-type lens remains transparent. Scale bar in top left panel=500 μm. **B**: While the lens sizes are comparable at P7 and P14 (p>0.05, n=12 lenses per group), the ephrin-A5^-/-^ lens becomes significantly smaller than the wild-type counterpart at P21 (p<0.05, n=12 lenses per group). **C**: Weights of the wild-type and ephrin-A5^-/-^ lenses are comparable at each of the stages. n=12 lenses per group. **D**: No differences are observed in posterior suture formation between both groups as both groups show the classical Y-suture formation. However, fiber cells appear more disorganized in the ephrin-A5^-/-^ lenses. Scale bar=50 μm.

**Figure 2:**
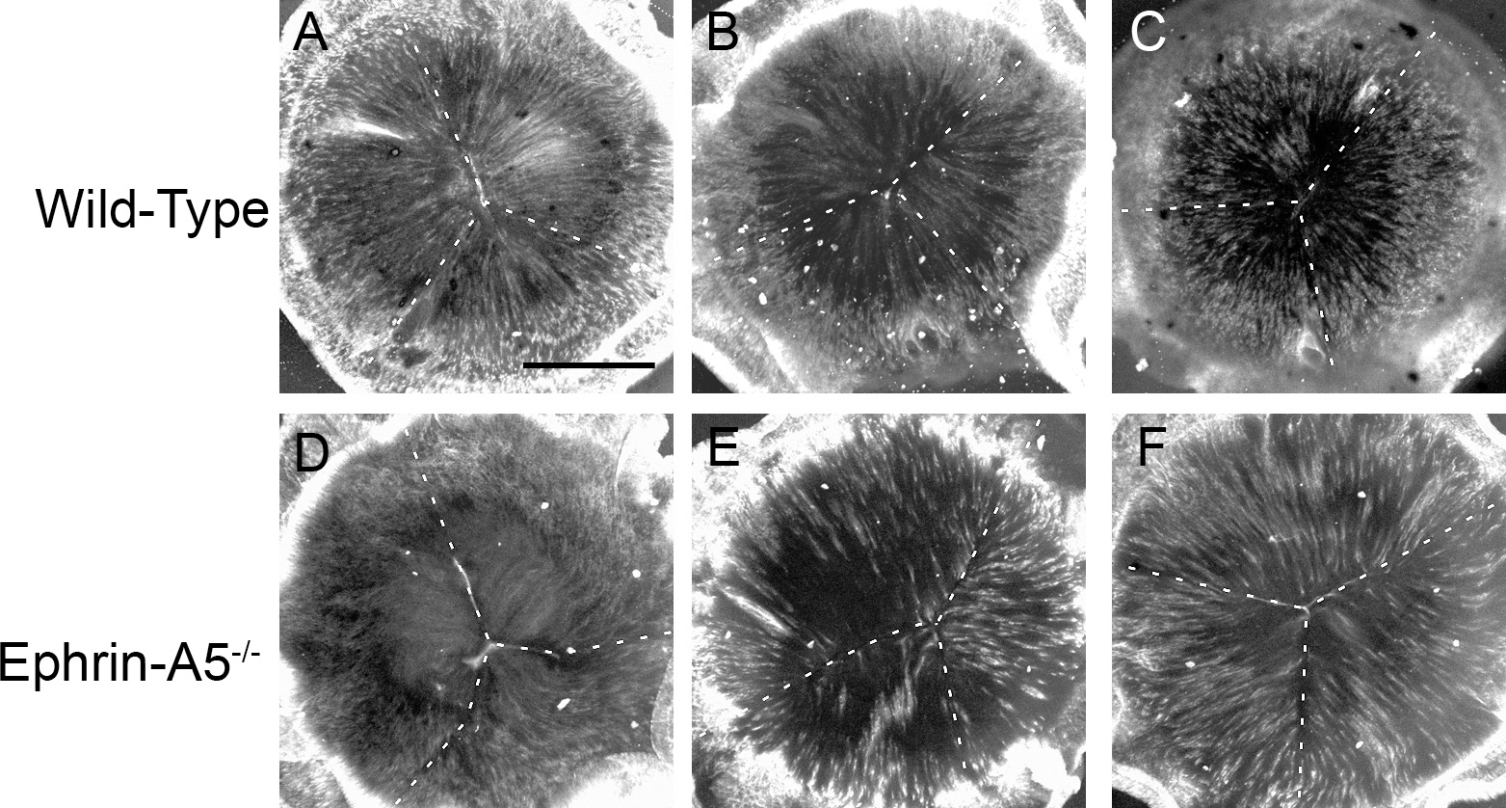
Low magnification posterior suture analysis shows no disruption of Y-shaped suture formation in the ephrin-A5^-/-^ lens. **A**-**F**: Wild-type lenses (**A**-**C**) display a Y-shaped suture structure with highly organized and packed fiber cells. Ephrin-A5^-/-^ lenses (**D**-**F**) also display a Y-shaped suture structure similar to wild-types. However, fiber cell organization and packing are disrupted in these lenses. Scale bar=500 μm.

Our previous studies have showed that ephrin-A5^−/−^ mice develop severe lens deficits, ultimately resulting in lens degeneration and cataract formation [32]. However, the cellular mechanisms underlying the cataract phenotype remained to be elucidated. We first asked how these changes affected the gross morphology of ephrin-A5^−/−^ lenses (Figure 1). To determine the refractive properties of wild-type and ephrin-A5^−/−^ lenses, eyes were enucleated and lenses were imaged under warmed media over a mesh grid. Imaging at postnatal day 7 (P7) and P14 showed no distinct alteration overall in lens morphology or light refraction between wild-type and ephrin-A5^−/−^ lenses. However, by P21, a dense opacity in the ephrin-A5^−/−^ lens had become visible, while the wild-type controls remained transparent (Figure 1A). When comparing lens diameters of wild-type versus ephrin-A5^−/−^ animals, no significant differences were observed until P21, at which point ephrin-A5^−/−^ lenses were found to be slightly but statistically significantly smaller than wild-type controls (Figure 1B). No significant differences in lens weight were observed between the two groups at any of the early postnatal stages (Figure 1C). Analysis of posterior suture formation in P14 lenses before cataract formation indicates that the Y-shaped structure was present in wild-type and ephrin-A5^−/−^ lenses (Figure 1D and Figure 2). However, while the suture shape appears normal, the organization of ephrin-A5^−/−^ lens fiber cells appears to be altered (Figure 2). These observations indicate that major lens deformities in the ephrin-A5^−/−^ animal occur in postnatal stages.

### Deformations in lens structure in the ephrin-A5^−/−^ postnatal eyes

**Figure 3:**
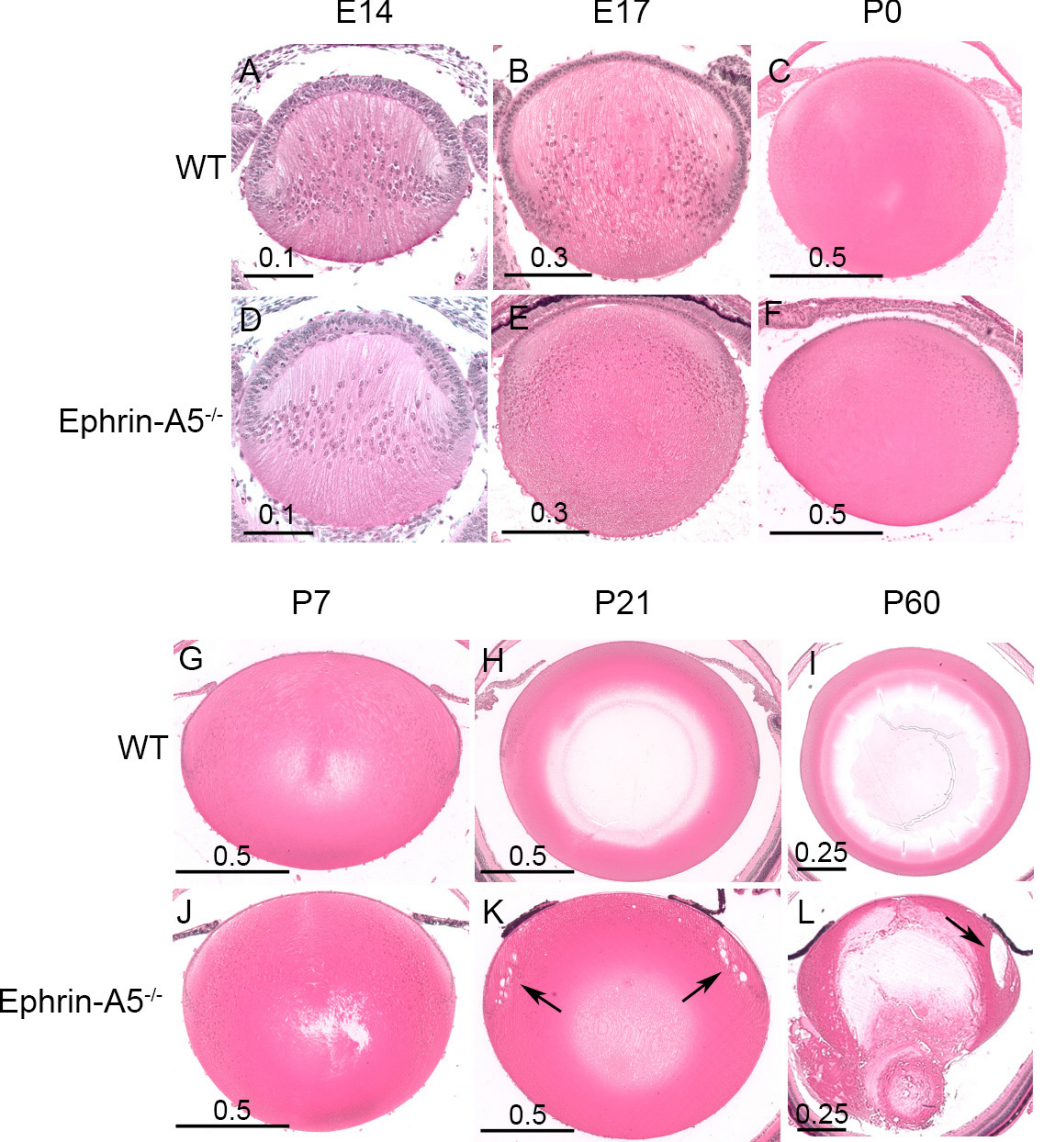
Deformations in the structure of ephrin-A5^-/-^ lenses occur in postnatal eyes. **A**-**F**: Embryonic development of wild-type (WT; **A**-**C**) and ephrin-A5^-/-^ (**D**-**F**) lenses appear similar, with no abnormalities observed in the early ephrin-A5^-/-^ lens. Scale bars in mm. **G**-**L**: While wild-type lenses (**G**-**I**) show no deformities in postnatal stages, ephrin-A5^-/-^ lenses (**J**-**L**) display noticeable lens deficits by P21 with the presence of vacuoles around the lens bow (compare **H** and **K**, see arrows). The deficits become progressively more severe, as larger vacuoles and complete posterior lens rupture is observed by P60 (Compare **I** and **L**, see arrow). Scale bars in mm.

We next set out to determine the developmental time in which lens aberrations occurred in the ephrin-A5^−/−^ mouse by analyzing histological sections of wild-type and ephrin-A5^−/−^ eyes from several embryonic and postnatal stages (Figure 3). Sections of embryonic and newborn ephrin-A5^−/−^ lenses revealed no clear morphological differences in comparison to wild-type controls, indicating that the overall development of ephrin-A5^−/−^ lenses during embryogenesis was normal (Figure 3A-F). However, analysis of ephrin-A5^−/−^ lenses at postnatal stages revealed the presence of lens deformities (Figure 3G-L). Lens deficits were observed in some mice as early as P6 and easily identified by P21 with the formation of large vacuoles near the lens bow region (compare Figure 3H,K, see arrows). These lens abnormalities were exacerbated in later stages, as complete lens degeneration was observed in the ephrin-A5^−/−^ lens by P60 (compare Figure 3I-L). Together, these observations indicate that the integrity of the ephrin-A5^−/−^ lens structure begins to fail in the postnatal stages.

### Disruption of lens fiber cell organization in the ephrin-A5^−/−^ lens

**Figure 4:**
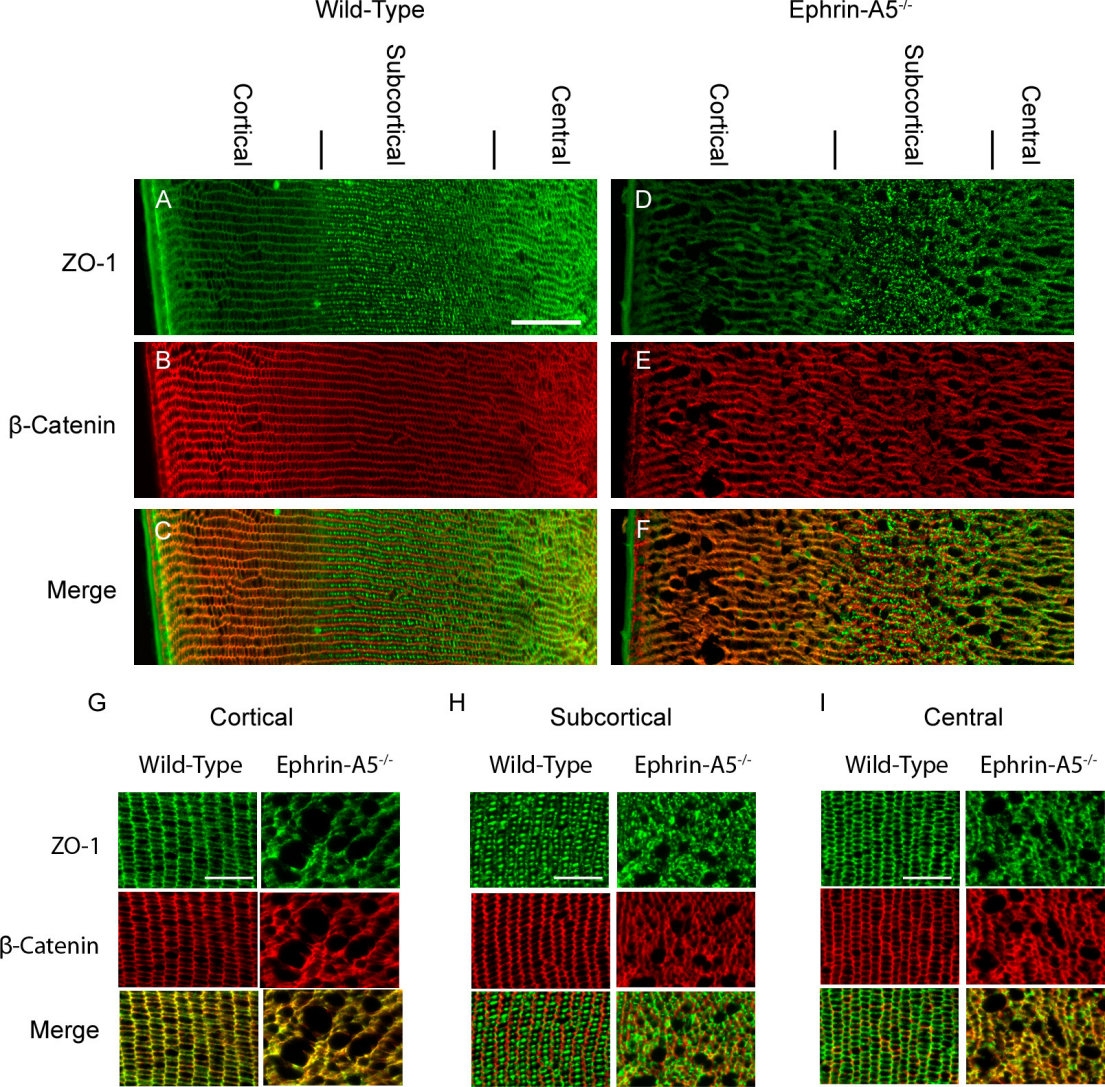
Distinct alterations in cell shape are observed in the ephrin-A5^-/-^ lens fiber cell layers. **A**-**F**: Wild-type (**A**-**C**) and ephrin-A5^-/-^ (**D**-**F**) P21 lenses are labeled for ZO-1 (**A** and **D**) and β-Catenin (**B** and **E**) to delineate cell borders or to distinguish distinct lens fiber areas. Disruptions in fiber cell organization are observed in the ephrin-A5^-/-^ lens. Scale bar = 100 μm. **G**-**I**: Disorganization of the fiber cells in the ephrin-A5^-/-^ lens are observed in all fiber cell regions, including the cortical (**G**), subcortical (**H**), and central (**I**) areas. Scale bar=50 μm.

**Figure 5:**
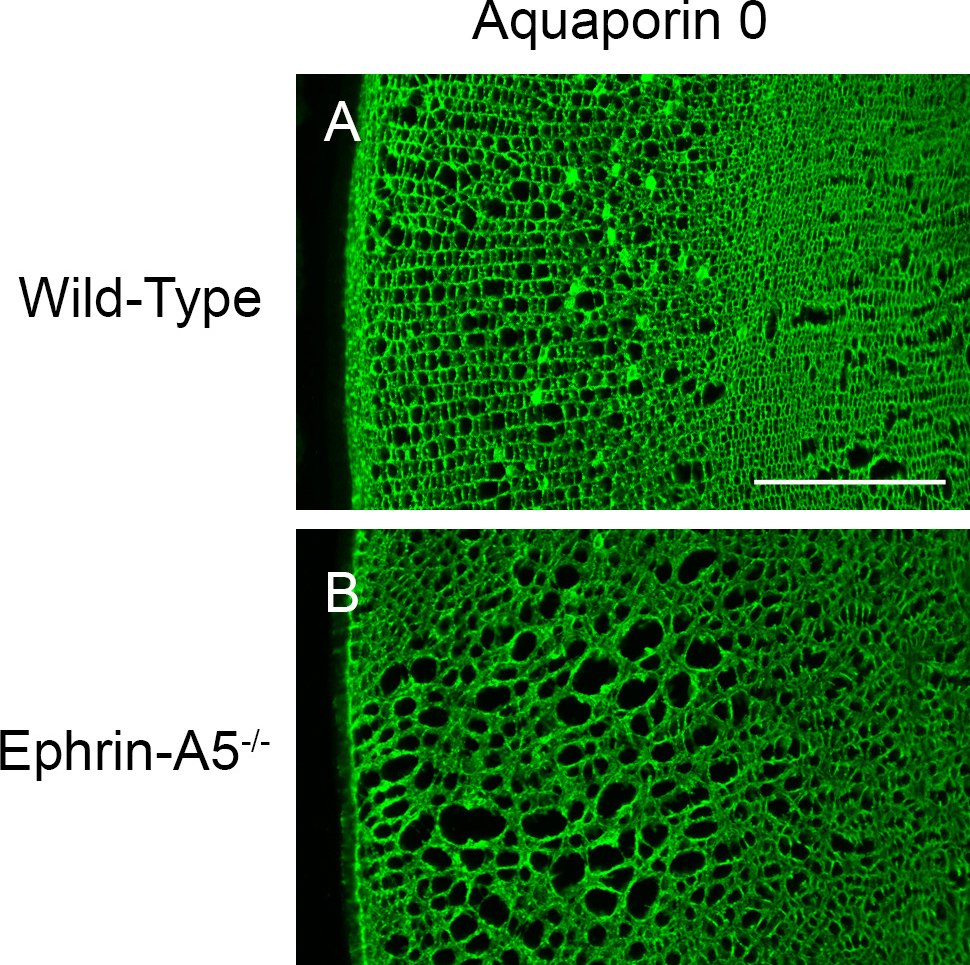
Expression of Aquaporin 0 in the ephrin-A5^-/-^ lens is observed along the cell membranes. **A** and **B**: In both wild-type (**A**) and ephrin-A5^-/-^ (**B**) lenses, Aquaporin 0 (AQP0) is found throughout the membranes of mature fiber cells. Scale bar=200 μm.

Our initial morphological analysis of ephrin-A5^−/−^ lenses revealed major alterations in fiber cell organization. However, the specific nature of these deficits remained to be elucidated, as changes in the regulation of the lens fiber cells, epithelium, or both could be resulting in vacuole formation in the mutant lenses. As a result, we set out to determine whether alterations of fiber cell organization were responsible for cataract formation in ephrin-A5^−/−^ animals. P21 lenses were cryosectioned coronally and immunostained for various markers, including the adherens junction molecule β-catenin and the tight-junction protein ZO-1, to delineate fiber cell borders and regions (Figure 4). Wild-type lens fiber cells were arranged in organized rows, with ZO-1 expression displaying distinct cortical, subcortical, and central regions (Figure 4A-C). Ephrin-A5^−/−^ lens fiber cells also showed the distinct ZO-1 layers; however, the fiber cells in these lenses were in disarray, with severe alterations being observed in the fiber cell shape (Figure 4D-F). Fiber cell disorganization was observed throughout the entirety of the fiber cell layers, as the cortical, subcortical, and central regions all exhibited a loss of organization (Figure 4G-I). Expression of the water channel protein AQP0 was also examined in wild-type and ephrin-A5^−/−^ lenses (Figure 5). AQP0 in the ephrin-A5^−/−^ lens was observed along the membranes of lens fiber cells with no distinct alterations from wild-type lenses except those that reflect the changes in cell shape (Figure 5A,B). Together, these findings indicate that while the overall differentiation of the ephrin-A5^−/−^ fiber cells is maintained, the organization of ephrin-A5^−/−^ fiber cells is severely disrupted.

**Figure 6:**
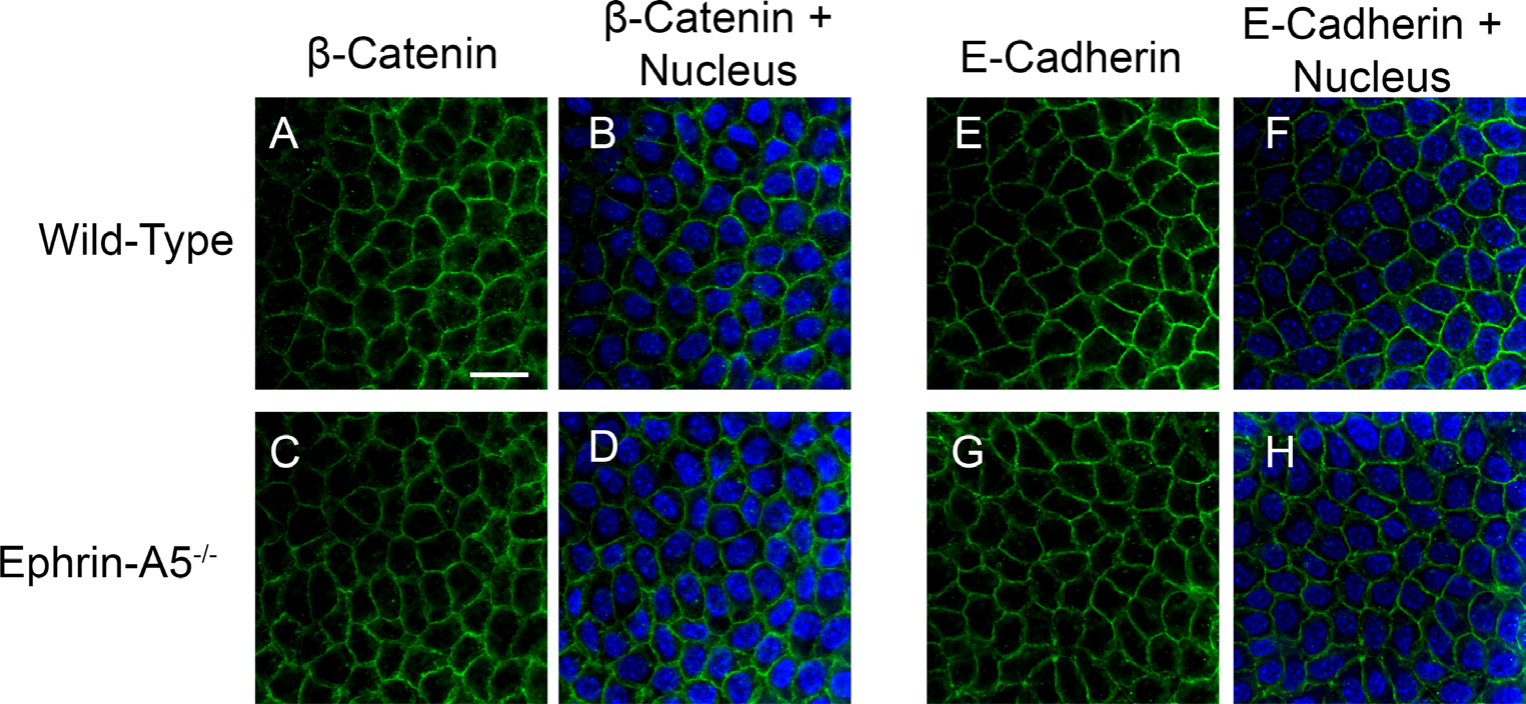
The lens epithelial regions appear undisturbed in ephrin-A5^-/-^ animals. **A**-**H**: No distinct differences in cellular morphology or adherens junction protein expression of β-catenin (**A**-**D**) and E-cadherin (**E**-**H**) are observed between the wild-type (**A** and **B**, **E** and **F**) and ephrin-A5^-/-^ (**C** and **D**, **G** and **H**) lenses in these regions. Scale bar=20 μm.

While alterations in lens fiber cell organization were observed in the ephrin-A5^−/−^ lenses, these disruptions could be a result of initial defects in the lens epithelium. A previous study reported major alterations of the adherens junction complex in the lens epithelium in ephrin-A5^−/−^ mice [31]. We therefore asked whether the lens epithelium is altered in these mutant mice and whether that may contribute to cataract formation (Figure 6). To determine whether any noticeable structural changes were present in the lens epithelium, lens epithelial explants of P21 wild-type and ephrin-A5^−/−^ lenses were stained for the adherens junction proteins β-catenin (Figure 6A-D) and E-cadherin (Figure 6E-H) to delineate lens epithelial cells. No differences were observed between the wild-type and mutant lens epithelium in regards to morphology or adherens junction expression (Figure 6). In addition, sagittal sections through wild-type and ephrin-A5^−/−^ lens epithelia were analyzed for β-catenin or E-cadherin expression. As with the epithelial explant analysis, no differences were observed between the two groups (data not shown). These results indicate that the alterations observed within the ephrin-A5^−/−^ lens are a result of defective lens fiber cell structures.

### CP49 status does not affect cataract formation in ephrin-A5^−/−^ mice

**Figure 7:**
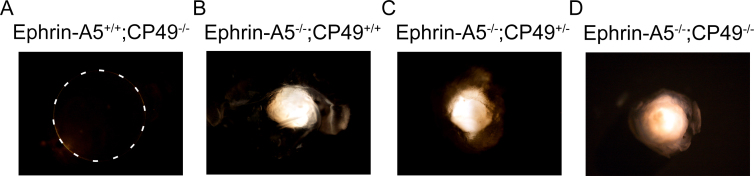
The status of CP49 does not affect ephrin-A5^-/-^ cataract formation. **A**-**D**: Ephrin-A5^+/+^ lenses with the CP49 mutation (Ephrin-A5^+/+^;CP49^-/-^) appear transparent (**A**, lens is denoted by dotted line), while ephrin-A5^-/-^ lenses, regardless of the status of CP49, display cataract formation (**B**-**D**). Lens deformations were observed in 100% of the ephrin-A5^-/-^;CP49^+/+^ lenses (n=4), 73% of the ephrin-A5^-/-^;CP49^+/-^ lenses (n=11), and 83% of the ephrin-A5^-/-^;CP49^-/-^ lenses (n=6).

The wild-type and ephrin-A5^−/−^ mice had been bred under a mixed background of C57BL/6, S129, and CD-1. Mice under the S129 background have been previously found to have deficiencies in the lens-specific intermediate filament protein CP49 [40,46]. We therefore analyzed whether the status of CP49 affected the formation of cataracts in ephrin-A5^−/−^ mice (Figure 7). Ephrin-A5^+/+^ mice with the CP49 mutation at P60 (Ephrin-A5^+/+^;CP49^−/−^) were found to be transparent with no observable light obstruction (Figure 7A), while ephrin-A5^−/−^ mice with wild-type, heterozygous, or homozygous mutant CP49 all developed cataracts at similar frequencies (Ephrin-A5^−/−^;CP49^+/+^: 100% [n=4]; Ephrin-A5^−/−^;CP49^+/−^: 73% (n=11); Ephrin-A5^−/−^;CP49^−/−^: 83% (n=6), Figure 7B-D). Together, this evidence suggests that the cataracts observed in ephrin-A5^−/−^ mice occur independently of CP49.

### Expression of ephrin-A5 and several Eph receptors found throughout the developing lens

**Figure 8:**
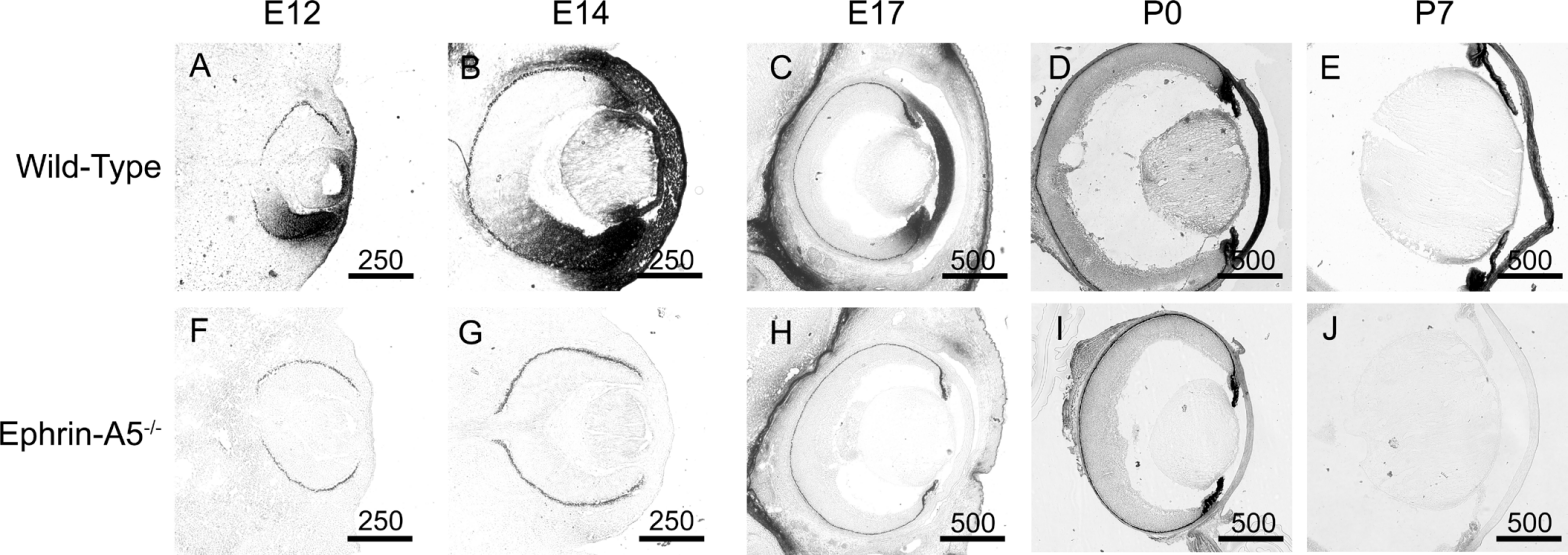
Ephrin-A5 is expressed extensively within the developing eye. **A**-**E**: EphA5-AP staining shows significant expression of ephrin-A ligand in the wild-type eye as early as E12 and persists through postnatal ages. Scale bars are in micrometers. **F**-**J**: Little to no EphA5-AP staining is observed in the ephrin-A5^-/-^ eye indicating that ephrin-A5 is the major ephrin-A ligand expressed in the eye. Scale bars are in micrometers.

We have found that ephrin-A5 plays a critical role in fiber cell maintenance in lens development. However, the timing and location of this factor in the developing lens have not been well characterized. To determine whether the spatial and temporal features of deficiencies in ephrin-A5 corroborate with the expression of the gene, we examined the localization of ephrin-A5 throughout murine eye development (Figure 8). Wild-type and ephrin-A5^−/−^ animals at several pre- and postnatal stages were sectioned and stained with EphA5-AP, a receptor for ephrin-A ligands. EphA5-AP staining was observed as early as embryonic day 12 (E12) in the wild-type eye, as expression was observed in both the retina and lens, with continued expression in the eye through P7 (Figure 8A-E). In contrast, the ephrin-A5^−/−^ eye showed little staining with EphA5-AP (Figure 8F-J).

**Figure 9:**
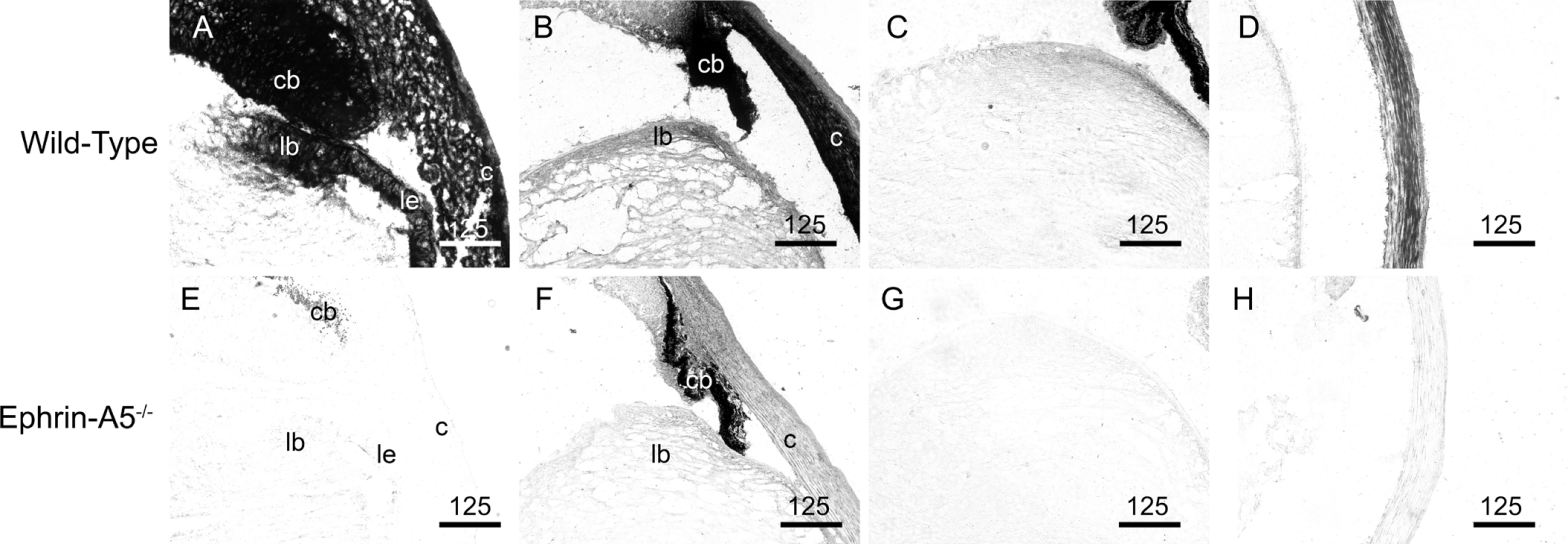
Ephrin-A5 expression is observed in several parts of the developing eye. **A** and **E**: EphA5-AP staining is observed in the lens epithelium (le), lens bow (lb), cornea (c), and ciliary body (cb) in the E14 wild-type eye while absent in the ephrin-A5^-/-^ animal. **B** and **F**: Expression of ephrin-A ligands are maintained in the wild-type at P0 though in lower levels in comparison with earlier embryonic stages, while remaining absent in the ephrin-A5^-/-^ eye. **C** and **G**: Ephrin-A ligand expression is observed in the lens bow region of P7 wild-type mice while absent in ephrin-A5^-/-^ mice at the same age. **D** and **H**: High levels of ephrin-A ligand is observed in the cornea of wild-type mice at P7 and not present in ephrin-A5^-/-^ mice at the same age. Scale bars are in micrometers.

EphA5-AP staining in wild-type tissues was particularly prominent in several parts of the eye (Figure 9). At E14, expression of ephrin ligands was observed in the lens bow, lens epithelium, ciliary body, and cornea (Figure 9A). Continued expression was observed in these areas at P0 and P7 (5C and D). However, levels of EphA5-AP staining in the lens were reduced during later developmental stages and further reduced at postnatal stages as time progressed, with little expression observed in the lens by P21 (data not shown). The robust staining of EphA5-AP in the wild-type eyes and absence of detection in the ephrin-A5^−/−^ eyes (Figure 9E-H) confirms ephrin-A5 to be the major ephrin-A ligand in the developing murine eye.

### Expression of the EphA2 receptor in the developing lens

**Figure 10:**
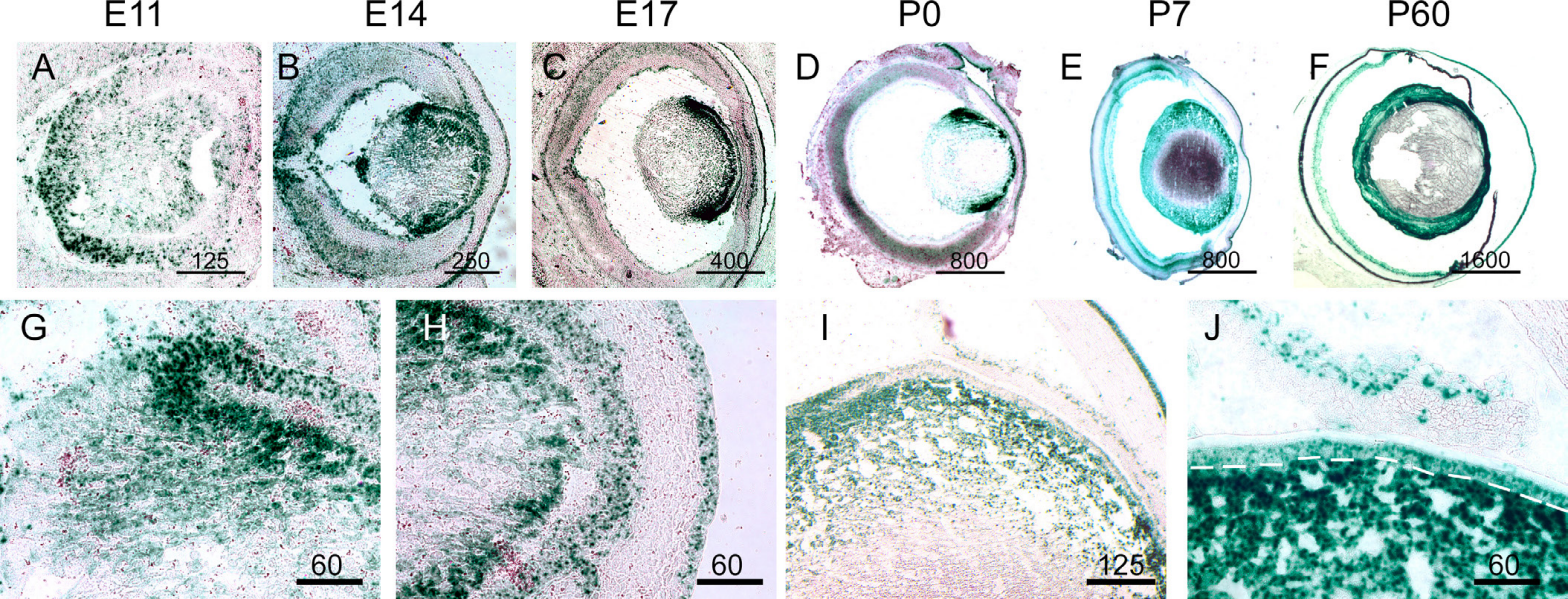
EphA2 is expressed throughout the developing lens. **A**-**F**: EphA2LacZ/+ tissue was reacted with X-Gal to detect EphA2 expression in the developing eye. Staining is observed in the E11 lens and found throughout lens development in subsequent embryonic and postnatal stages. Scale bars are in micrometers. **G** and **H**: At E14, expression is observed in the lens fiber cells near the bow and lens epithelium (**G**). Extensive expression is also observed and near the junction between fiber cells and epithelium (**H**). **I** and **J**: At P7, EphA2 is expressed in the outer lens fiber cell regions (**I**) as well as in the lens epithelium (**J**, epithelium is delineated by white dotted line). Scale bars are in mictrometers.

Ephrin activity is mediated through its interactions with Eph receptors. Previous reports have indicated that EphA2 is important in lens development, as mutations in this Eph receptor are known to result in cataractogenesis [33,35-38]. We therefore analyzed the expression profile of EphA2 at various developmental stages. Similar to ephrin-A ligand localization in the lens, EphA2 localization is observed early in lens development, being detected in the presumptive lens as early as E11 (Figure 10A). This expression continues through lens development and well into adulthood, with expression in the lens being observed as late as P60 (Figure 10B-F). At E14, EphA2 expression is observed in both the lens epithelium and in the lens fiber region near the bow region similar to that observed in the ephrin-A ligand expression (Figure 10G). In addition, levels of EphA2 are also seen at the junctions between the lens fibers and epithelium in the anterior portion of the lens (Figure 10H). At P7, expression of EphA2 is still observed in both the lens fiber subcortical region and in the lens epithelium (Figure 10I,J). Together, these data indicate that strong expression of EphA2 is present throughout lens at embryonic and postnatal periods.

## Discussion

This study has further characterized and detailed the cataract phenotype in ephrin-A5^−/−^ mice. In addition, we have found that ephrin-A5 and EphA2, Eph family molecules known to play significant roles in lens development, are expressed throughout the lens starting during early prenatal development and are expressed at postnatal stages with similar localization.

The ephrin-A5^−/−^ mice used for this study are under a mixed background of C57BL/6, S129, and CD-1 strains, while the EphA2^LacZ/LacZ^ mice are under an FVB/NJ background. Other studies on the effects of ephrin-A5 on the lens using mice under mostly C57BL/6 background have found alterations in the lens epithelium with minimal alterations in the lens fiber cells and severe changes in the lens epithelium [31]. In our current study, we observed major alterations in lens fiber cell organization but observed no disruptions in the lens epithelium. Differences in mouse genetic background may explain the difference in observed phenotype, as differences in cataract phenotypes were also observed in different EphA2^-/-^ strains [33,34]. One known mutation in S129 strains affecting the lens is the deletion of the intermediate filament protein, CP49 [40,46]. Our own observations have found that regardless of the status of CP49, ephrin-A5^−/−^ mice under this mixed background still develop cataracts indicating that the CP49 protein may not directly affect the cataracts observed in ephrin-A5^−/−^ mutants. However, this does not discount other differences between mouse strains that may be contributing to the differences in cataract formation.

EphA2^−/−^ mice develop congenital cataracts in a manner similar to ephrin-A5^−/−^ mice, developing subcapsular vacuoles leading to lens opacity and rupture [33]. In the current study, EphA2 expression in the lens during development was found in similar locations with ephrin-A5, including the lens fiber regions near the bow and the lens epithelium. Additionally, a significant amount of EphA2 expression was also observed in the anterior regions of the fiber cell layer near the junction with epithelial cells. Based on our expression data and previous studies, the Eph family may have additional roles in lens development in addition to the maintenance of fiber cell organization. EphA2^−/−^ lenses have been previously reported to have sutural deficits and form epithelial lesions [34]. Together, this may indicate that EphA2 plays a role in the formation of epithelial and fiber cell junctions.

While ephrin-A5 and EphA2 are expressed in the lens at early stages, the role of ephrins in prenatal lens development, if any, remains unclear. The embryonic development of the ephrin-A5^−/−^ lens is grossly normal, as abnormalities were not observed in the ephrin-A5^−/−^ lens until early postnatal stages. One possibility for this lack of phenotype in early development is that the need for highly ordered and structured fiber cells may not be required in the embryonic lens. In normal lens development, primary lens fiber cell layers are polygonal and disorganized, whereas the secondary fiber cell layer are highly regular flattened hexagonal cells [1,2]. Another possibility is that the lack of phenotype at early developmental periods may be dependent on other ephrin ligands at the embryonic stages that are insufficient or absent in postnatal periods. Specifically, other Eph-ephrin interactions that are not detected by EphA5-AP staining, including the B-class ephrins, may be playing concurrent roles in early lens development and preserving the majority of developmental activity that this family of molecules plays during early development. It may also be possible that the major roles of ephrin-A5 regulation of the lens occur in early stages of development and not during the postnatal periods, with its absence during these critical periods making the lens susceptible to alterations during maturation ultimately leading to cataracts. This may explain the prominent ephrin-A5 lens expression at earlier embryonic stages of ocular development and reduced levels seen in postnatal stages.

Though both ephrin-A5 and EphA2 share a similar expression profile in the lens, the ephrin-A5 and EphA2 mutant mouse models indicate a distinct difference in the timing of cataract onset. Ephrin-A5^−/−^ animals have noticeable lens deficits as early as P6 and become opaque by P21, while EphA2^−/−^ animals develop lens deficits at 1 month of age and cataracts by 5 months [33]. One possible explanation for this difference is the compensation of other EphA receptors in the mature lens; several Eph receptors are present in the lens (data not shown) and may play compensatory roles in the absence of EphA2. In contrast, EphA5-AP staining detected high levels of ephrin ligand expression in the wild-type lens but very little in the ephrin-A5^−/−^ lens, indicating that ephrin-A5 is the major A-class ligand in the mature lens. The lack of compensation by other ephrin-A ligands may therefore result in an earlier cataract phenotype in the ephrin-A5^−/−^ lens, while the presence of other distinct EphA receptors aside from just EphA2 may cause a delay in the lens phenotype. The later onset of the phenotype by the EphA2^−/−^ animals may also be due to the animal background of the ephrin-A5^−/−^ mice and the EphA2^−/−^ animals, as variability of the cataract phenotype has been documented in other studies [33,34].

While the gross morphology of the ephrin-A5^−/−^ lens appeared normal in the early postnatal stages, the size of the lens in ephrin-A5^−/−^ was found to be slightly smaller than wild-type controls at P21, a stage during which cataract formation is first apparent. One possibility is that lens fiber cells have started to degenerate at this time, as ephrin-A5^−/−^ lenses before cataract formation appear similar in size to wild-type ones, although the mutation may also affect lens growth. Fiber cell packing during the differentiation of epithelial cells may also be affected in the ephrin-A5^−/−^ lenses, leading to further problems in fiber cell organization and alignment. Future studies focusing on these aspects of lens fiber cell differentiation and organization may yield additional roles of ephrin-A5 in the maturation of the lens.

Our current study indicates that deficits in the ephrin-A5 lens are primarily in the lens fiber cell layers. Unlike the stereotypical lens fiber cell architecture, in which cells have uniformly elongated hexagonal shapes arranged in regular rows, lens fiber cells in ephrin-A5^−/−^ animals are disarrayed, with fiber cells in various orientations. In addition, large vacuoles form between fiber cells, contributing to the disorganization and eventual cataract formation of the ephrin-A5^−/−^ lens. The maintenance of lens fiber cell architecture is dependent in large part on the interactions between adhesion molecules and the organization of cytoskeletal elements. N-cadherin is the predominant cadherin molecule in the lens fiber cells regulating intercellular interactions, and has been found to be an important factor in the organization and packing of the differentiated cells [14,16,17,47]. We reported previously that ephrin-A5 has a major role in the regulation of N-cadherin localization in the lens fiber cells, and that ephrin-A5 activity has been found to affect interactions between N-cadherin and β-catenin, a regulator of the adherens junction [32]. The relationship between ephrins and the adherens junction have also been previously documented [48-53]. Future studies looking at the mechanisms by which ephrin-A5 and its putative Eph receptor regulates the adherens junction in the lens may yield a greater understanding of the role of ephrins in lens fiber cell regulation.

The deficits in the lens fiber cells of the ephrin-A5^−/−^ lens may result in additional consequences as a result of the disorganization in lens architecture. The uniform packing of lens fiber cells is particularly important to maintain proper lens circulation given the absence of vasculature [10,11]. Our present study has found that while localization of ZO-1, a protein involved with lens circulation and gap junction regulation, remains along the cell membrane in the ephrin-A5^−/−^ lens, the organization of these structures is highly disrupted. The correct regionalized localization of ZO-1 in the cortical, subcortical, and central lens areas implies that ephrin-A5 may have only an indirect impact on the regulation of gap junction proteins. However, the disorganization of the fiber cell layers may severely affect the circulation of nutrients throughout the lens, further contributing to cataract formation.

In summary, ephrin-A5 is the predominant ephrin-A ligand in the lens and is a critical regulator of lens fiber cell organization. Future studies elucidating the signaling events by which ephrins mediate this organization are likely to provide meaningful insightful regarding the organization of secondary lens fiber cells.
